# Evolution of intraspecific transcriptomic landscapes in yeasts

**DOI:** 10.1093/nar/gkv363

**Published:** 2015-04-20

**Authors:** Christian Brion, David Pflieger, Anne Friedrich, Joseph Schacherer

**Affiliations:** Department of Genetics, Genomics and Microbiology, University of Strasbourg, CNRS, UMR7156, Strasbourg, France

## Abstract

Variations in gene expression have been widely explored in order to obtain an accurate overview of the changes in regulatory networks that underlie phenotypic diversity. Numerous studies have characterized differences in genomic expression between large numbers of individuals of model organisms such as *Saccharomyces cerevisiae*. To more broadly survey the evolution of the transcriptomic landscape across species, we measured whole-genome expression in a large collection of another yeast species: *Lachancea kluyveri* (formerly *Saccharomyces kluyveri*), using RNAseq. Interestingly, this species diverged from the *S. cerevisiae* lineage prior to its ancestral whole genome duplication. Moreover, *L. kluyveri* harbors a chromosome-scale compositional heterogeneity due to a 1-Mb ancestral introgressed region as well as a large set of unique unannotated genes. In this context, our comparative transcriptomic analysis clearly showed a link between gene evolutionary history and expression behavior. Indeed, genes that have been recently acquired or under function relaxation tend to be less transcribed show a higher intraspecific variation (plasticity) and are less involved in network (connectivity). Moreover, utilizing this approach in *L. kluyveri* also highlighted specific regulatory network signatures in aerobic respiration, amino-acid biosynthesis and glycosylation, presumably due to its different lifestyle. Our data set sheds an important light on the evolution of intraspecific transcriptomic variation across distant species.

## INTRODUCTION

In the phenotypic diversity of a species, variation in transcription and abundance of mRNA is one of the first ties linking genetic mutations to complex traits. How the expression of a gene is controlled or triggered due to environmental changes and mutations depends on its function and evolutionary history. Amenable to the laboratory, the yeast *Saccharomyces cerevisiae* has been a good model to decipher the factors controlling gene expression (1).

Observing expression variation in response to growth conditions in this model yeast has allowed researchers to assess the resulting shifts in metabolism (2). Gene expression responses have been described during aerobic growth (3), meiosis (4), stresses (5,6) and in the fermentation and industrial production context (7). These analyses have also allowed improved functional gene annotation (8,9).

In order to understand how genetic background affects gene expression, the transcriptomic profiles of many strains obtained in the same conditions could allow for the observation of intraspecific expression variation (10–12). These changes could be linked to other phenotypes and ultimately provide a better overview of the diversity within a species (11). The majority of the genes are organized in modules of co-expression, which are related to highly ramified regulatory networks of genes involved in the same pathway. Indeed, even across distinct isolates, it has been shown that genes involved in the same processes are likely to display similar patterns of variation (11,12). This indicates that mutations between strains mainly have basal impacts that affect whole regulatory networks.

Focusing on a model species has allowed for the development of highly accurate tools dedicated to functional analysis of data but this does not provide a broad enough view of variation among different species and may lead to the incorrect assignment of potentially unique behaviors to the entire phylum. For instance, *S. cerevisiae* displays specific characteristics linked to its domestication such as low genetic diversity between isolates and clustering according to industrial processes (13,14). It is now necessary to expand gene expression analyses to other species, which has already been attempted in some studies. The transcriptomic responses to different growth conditions in *Saccharomyces bayanus* had been compared to those of *S. cerevisiae* (15), and led to the description of new gene functions. In *Candida glabrata*, RNA sequencing allowed for the investigation of responses in transcription to pH variations and has led to novel gene descriptions (16). The comparison of the expression levels across four *Saccharomyces sensus stricto* species has allowed the establishment of the link between expression divergence and the presence of the TATA box (17). The more distantly related pathogenic yeast *Candida albicans* has also been thoroughly studied to link infectious traits to expression (18,19). In an inter-species study, Thompson *et al*. focused on deciphering the evolution of co-expression modules across 15 *Ascomycota* species (20,21). Taking into account gene duplication, they proposed mechanisms potentially underlying conservation and changes in gene networks.

These studies compared evolution of gene expression across species but none have taken into account intraspecific variation. Indeed, changes in expression between isolates reflect the mechanisms of adaptation and evolution within a species, which is likely different among yeast lineages. It is now crucial to expend transcriptomic surveys to other strain collections of species more distantly related to the conventional model yeast *S. cerevisiae* in order to decipher how regulation mechanisms and types of genetic controls have evolved.

Therefore, we undertook the investigation of gene expression variation in the yeast *Lachancea kluyveri*. This species possesses many characteristics that make it a powerful alternative model organism for transcriptomic studies. The genome of the reference strain CBS 3082 has already been sequenced and annotated, confirming the identity of *L. kluyveri* as a protoploid species (22). This will allow us to explore the evolutionary implications of the whole genome duplication (WGD) through comparative analyses and to describe the behavior of ohnologous genes.

This sequencing also identified *L. kluyveri* genes that have no orthologs in the model species, *S. cerevisiae*, with 302 genes that were only identified in closely related lineages and 155 unique to this species. Therefore, *L. kluyveri* displays an unusually high number (457) of uncharacterized open reading frames out of the 5278 that, along with 369 non-coding RNAs, are distributed across eight chromosomes for a total nuclear genome of 11.3 Mb (22).

Interestingly, the reference genome displays an intriguing compositional heterogeneity: a region of ∼1 Mb, covering almost the entire left arm of chromosome C (hereafter called Sakl0C-left). This particular region has an average GC content of 52.9%, which is significantly higher than the 40.4% global GC content found for the rest of the genome (23). The origin of this compositional heterogeneity was not well understood until recently. The survey of 28 natural isolates of this species allowed us to propose that this left arm of chromosome C was acquired by an introgression event (24). These 28 isolates cluster according their geographic origin and display high levels of diversity up to 2.6%, while *S. cerevisiae* harbors a maximum of ∼0.6%.

Therefore, the phylogenetic distance between *L. kluyveri* and *S. cerevisiae* is associated with interesting genomic differences, which could not be studied using a more closely related species, such as *Saccharomyces paradoxus*. Here, we employed an RNAseq analysis to characterize gene expression patterns and described the variation in gene regulation of 24 haploid *L. kluyveri* isolates. We compared these results to gene expression variation previously assessed in 22 *S. cerevisiae* strains (12), thereby focusing our analysis on the gene network variation that distinguishes *L. kluyveri* from *S. cerevisiae*. Moreover, the WGD undergone by *S. cerevisiae* ancestor and the chromosome C introgression in *L. kluyveri* provided the opportunity to decipher the impact of gene acquisition on intraspecific expression variation. We clearly demonstrate that recently acquired genes show lower expression levels, are associated with higher rates of variability and tend to be less integrated in large modules. Finally, using co-expression patterns, we associate putative biological processes with 36% of *L. kluyveri* uncharacterized or poorly annotated genes.

## MATERIALS AND METHODS

### Yeasts and growth conditions

**Figure 1.**
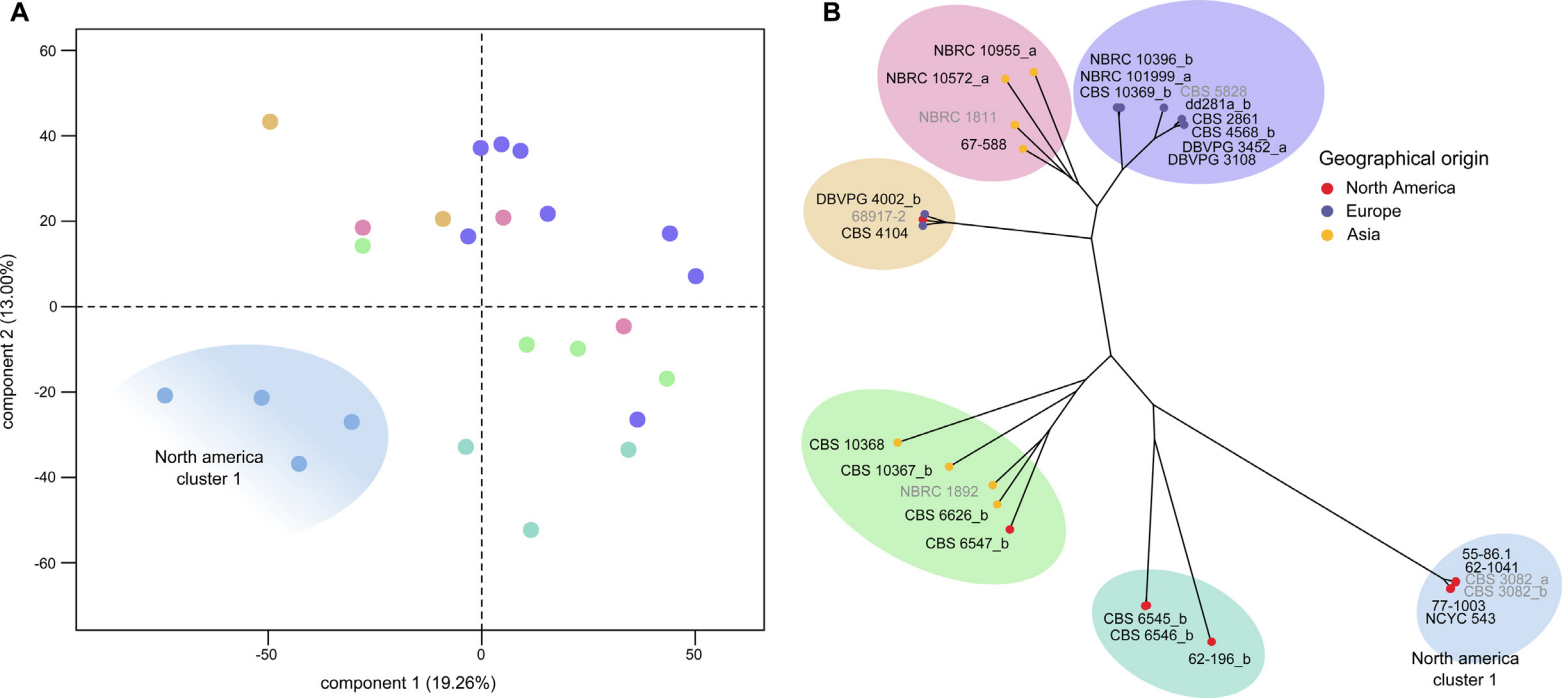
Global structure of the transcriptomic data across the collection of natural isolates. (**A**) PCA analysis of the global expression data, each dot corresponds to a strain, with color matching the phylogenetic cluster. (**B**) Neighbor-joining tree of the 28 sequenced strains of *L. kluyveri* based on SNP variants (adapted from (24)).

The expression analyses were performed over 24 strains out of the 28 strains previously sequenced (24). The 24 strains are listed in Figure 1B. The isolate CBS 3082 was used as a reference. To perform growth test, strains were plated on solid Yeast extract Peptone Dextrose (YPD) and allowed to grow for 1 to 3 days, then transferred to 30 ml of the test medium (liquid YPD) overnight at 30°C. The growth was followed by measurements at OD_600_.

### RNA sampling

The cells were sampled by filtration during the mid-exponential growth phase in YPD at 30°C at an OD_600_ between 0.250 and 0.350. For each strain, two cultures were used as biological replicates for the RNA analysis. After filtration, cells were frozen in liquid nitrogen and stored at −80°C. The RNA extraction was performed with hot aqua-phenol. Cells were treated with a lysis buffer (ethylenediaminetetraacetic acid 10 mM, sodium dodecyl sulphate 0.5%, TricHCl 10 mM pH7.5) and aqua-phenol (MP AQUAPHEO01) at 65°C for 1 h. After a thermal shock of 10 min in ice, the aqueous phase was extracted by centrifugation for 5 min at 12 400 rpm. Traces of protein and cell fragments were removed by a second centrifugation (5 min at 12 400 rpm) with chloroform in a Phase Lock Gel: 5prime 2302830 (PLG) tube. The nucleic acids in the supernatant were precipitated with 70% ethanol and 3-M sodium acetate overnight. After a centrifugation for 10 min at 12 400 rpm at 4°C, the pellet was resuspended for purification on the RNeasy membrane (Qiagen 74104) and a DNase treatment (Invitrogen 18068-015) was completed. Quality was verified by gel migration and spectrophotometry (NanoDrop ND-1000).

### RNA sequencing and data processing

RNA samples were sequenced at Gene Core Facility—EMBL (Heidelberg, Germany). The libraries were prepared for multiplex Illumina HiSeq2000 sequencing using 50-bp non-oriented single end reads. Raw reads were analyzed using FastQC report and cleaned with CutAdapt (25). Overrepresented sequences were removed if identified as rRNA or Trueseq adaptor contaminants. Low quality reads were filtered and trimmed (Phred score inferior to 25 and size less than 40 nt were discarded). The reads were then aligned with Tophat v2.0.8b (26) to the reference genome (CBS3082) for which Single-Nucleotide Polymorphism (SNPs) were inferred for each strain. This allowed for an alignment rate from 96.3 to 98.6% with the total number of mapped reads ranging from 8 to 25 million. We used the Génolevure annotation file of CBS3082 to define the intragenic regions and HTseq-count (27) to obtain the number of reads for each region. Between 87 and 97% of the reads that were mapped fell in the annotated regions. Raw data are available on extractable nuclear antigen under accession number PRJEB8449.

Before normalization, regions with low coverage (number of reads lower than 32 (2^5^) for all samples) were discarded. Normalization was completed on R (28) with bioconductor DEseq2 allowing for comparison of data between each sample. Because of a bias on Sakl0C-left during library preparation due to the change in GC content, this region was normalized separately. Base 2 logarithms were then used to assess expression level. The lowest Spearman correlation coefficient between biological replicates is 0.978, indicating good data stability. The averages of expression between replicates were then used for further analysis. In some strains, we observed an over-expression of chromosomes (strain NBRC 10396_b: chromosomes 1 and 3; strain 55-86.1: chromosomes 3 and 5; strain CBS 6545_b: chromosome 6; strain 77-1003: chromosome 8 (right arm) and chromosome 3) corresponding to instance of aneuploidies. We disregarded the expression data of genes located on these duplicated chromosomes.

### Comparison to expression in *S. cerevisiae*

We used the RNAseq expression data of 22 *S. cerevisiae* isolates published by Skelly *et al*. (12) that is publicly available (http://www.yeastrc.org/g2p/). Orthology between *L. kluyveri* and *S. cerevisiae* was determined based on *L. kluyveri* annotations (22). Annotation was completed according to the amino acid identity levels across the entire protein sequence, unless noted otherwise: ‘highly similar’ > 80%; ‘similar’ = 50–80%; ‘weakly similar’ = 30–50%; ‘some similarity’ > 50% (limited to a domain).

### Expression clustering analysis

To select genes specifically involved in significant clustering, a first analysis was performed on all data using the R hclust function (method complete, stats package). By permutation, we determined that a height threshold of 3 allowed us to expect less than 40 genes to cluster by chance (0.8%) in *L. kluyveri* and 150 in *S. cerevisiae* (2.6%). All genes forming a cluster with a height less than 3 were used in a second clustering analysis with Cluster 3.0 (complete linkage) and JavaTreeView to utilize an efficient GUI.

### Growth rate correlation

In cultures for cells sampling, the OD_600_ was followed, providing two growth rate values for each strain. For each gene, the Spearman correlation between growth rate and expression was calculated. Taking Bonferroni–Hochberg-corrected *P*-values lower than 0.05 gave a threshold of 0.6 for the correlation coefficient.

### Connectivity

Using R (28), the pair-wise Spearman correlation between genes expression levels was calculated. For each gene, we obtained the numbers of genes that were positively correlated. By permutation, we determined that when using a permissive threshold of 0.65 as correlation coefficient, only 14 genes (0.2%) have more than nine correlations due to chance in *L. kluyveri*, while 66 genes (1.2%) have more than nine correlations due to chance in *S. cerevisiae*.

### Bimodality

A *k*-means test was performed for the expression of each gene across all isolates with *k* = 2. The unbalanced clusters were disregarded by ignoring the results when the number of individuals in the smaller cluster was less than 4. By applying the test to 5000 random normal and Poisson distributions, we determined a significance threshold of 0.87 for the ratio of the sum of squares between clusters to the total sum of squares, for which no unimodal distribution was detected as bimodal by chance.

### Calculation of dN/dS values

The dN/dS ratios were calculated for each gene in all strains. Values were determined by running the YN00 model (PAML package, version 4.5 (29)) on the multiple sequence alignment of the coding regions of each protein-coding gene. Theses alignments were constructed by inferring polymorphic positions. For each gene, we used the average dN/dS values calculated across strains.

### Gene ontology term enrichment

Research of functional enrichment during clustering analysis, growth rate correlation and *S. cerevisiae*/*L. kluyveri* comparisons were performed using FunSpec with a significant threshold of 10^−5^ (30).

### Assignment of putative biological process to genes

From the connectivity analysis, we obtained, for each gene G, the list of co-expressed genes and retained only those demonstrating a similarity higher than 50% with their *S. cerevisiae* orthologs. By permutation, we determined that only ∼2.5% of genes are expected to display more than five correlated genes due to chance. From that list, we computed the highest gene ontology (GO) term enrichment, which was then assigned the gene G. We used topGO from the R bioconductor package, with the ‘classic’ algorithm and ‘Fisher’ statistic test (31) and used org.Sc.sgd.db as a database. By permutation, we determined that, at a *P*-value threshold of 10^−4^ for the enrichment score, the false discovery rate was lower than 10% (around 8%). We only kept the results for genes with four or more correlations to ensure that the enrichment was reliable.

## RESULTS

### Exploration of the transcriptomic landscape in *L. kluyveri*

We performed a transcriptomic analysis of 24 *L. kluyveri* isolates and obtained mRNA levels for the 5647 regions annotated in *L. kluyveri*. To have a better overview of the differential behavior among strains and to determine which strains displayed similar expression patterns, we performed a principal component analysis (Figure 1A). We observed that four strains were separated from the main group. These strains are closely related and all belong to a genetic cluster identified during the sequencing (diversity lower than 0.1%, Figure 1B, (24)). Concerning the 20 additional strains, no clear clustering can be identified, indicating a complex structure of changes in expression. The previously described genetic clusters of natural isolates have no echo on the overall transcriptomic behavior of strains, suggesting that the distant evolutionary history of strains has no major impact on expression variation.

We then considered the variation in mRNA abundance for each gene, which is dictated by transcription rate and degradation. There are 520 genes that display a maximum range of expression higher than 4 (log_2_(normalized read number)), indicating that they could reach a 16-fold change in transcription level between different genetic backgrounds. To identify GO term enrichment among the genes with high levels of variations (standard deviation over than 1.5), we used *S. cerevisiae* orthologs. Unfortunately, approximately half of the highly variable genes do not have *S. cerevisiae* orthologs. Those that do displayed significant GO term enrichment in transmembrane transport, including hexose transporters (*P* = 1.4 × 10^−10^) and flocculation (*P* = 3.6 × 10^−5^). We also observed, in this category, genes involved in fermentation (pyruvate decarboxylase, alcohol dehydrogenase) and pheromone response without significant enrichment. Conversely, searching for GO term enrichment for genes that display very low standard deviations (under 0.2) identified function associated with transcription (*P* = 8 × 10^−8^), mRNA processing (*P* = 2 × 10^−11^) and protein transport (*P* < 10^−14^). Consequently, genes with conserved transcription levels across isolates are mostly involved in gene expression and correspond to the core genome. Contrastingly, the largest variations in transcription concern genes related mainly to import and export processes that could be linked to potential means for adaptation to changes the environmental conditions.

### Evolution of expression highlighted by comparison with *S. cerevisiae*

**Figure 2.**
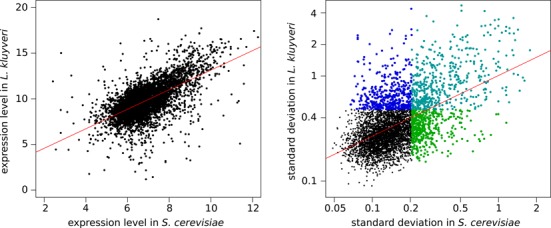
Comparison of transcription level (left) and variation (right) between *L. kluyveri* and *S. cerevisiae* orthologs. The transcription levels correspond to the mean of the transcript abundance across all isolates of each species. Colors on the variation plot correspond to the sets of genes we used for enrichment in order to identify the difference in transcription between *L. kluyveri* and *S. cerevisiae*. The standard deviation axes are in a log scale.

To obtain a better view of the characteristics regarding variation in expression of preduplicated yeast, we compared the plasticity of transcription for each *L. kluyveri*–*S. cerevisiae* ortholog pair. For all comparisons, we used previously published expression data from 22 *S. cerevisiae* strains (12). We first compared the average expression levels of each ortholog pair and observed a noisy (linear model R^2^ = 0.39) but significant (*P* < 2 × 10^−16^) correlation, despite the considerable genetic distance between the two species and the differences in conditions tested (Figure 2). This indicates that the transcript abundance was generally maintained for a fraction of genes, which is probably linked to the conservation of the molecular function of the genes that dictated the protein abundance.

We also investigated the sensitivity of gene expression to genetic background by comparing the standard deviation for each ortholog pair (Figure 2). We considered genes for which the standard deviation was above the 80% quantile (i.e. the 20% more variable genes) and focused on three type of behavior in ortholog pairs. First, we analyzed pairs that are highly variable among *S. cerevisiae* and *L. kluyveri* isolates (397 genes), which mainly included genes involved in transmembrane transport (*P* = 8.7 × 10^−11^), the carbohydrate metabolic process (*P* = 2.2 × 10^−8^), the alcohol metabolic process (*P* = 3.3 × 10^−6^) and the tricarboxylic acid cycle (*P* = 5.6 × 10^−6^). Second, we looked at pairs that were highly variable in *L. kluyveri* isolates but not in *S. cerevisiae* (416 genes). Among them, we found enrichment for sulfate assimilation and methionine biosynthesis (*P* = 1.2 × 10^−5^) as well as inosine biosynthesis (*P* = 9.9 × 10^−5^). Finally, we examined pairs that were highly variable among *S. cerevisiae* but not *L. kluyveri* isolates (447 genes). This last set comprises genes involved in adenosine triphosphate (ATP) synthesis/proton transport (*P* = 10^−9^), mitochondrial translation (*P* = 1.9 × 10^−9^), the cytochrome c/electron transport chain (*P* = 4.2 × 10^−7^) and nicotinamide adenine dinucleotide biosynthesis (*P* = 1.4 × 10^−6^). Interestingly, we observed that transcription of the genes involved in respiration tended to be less variable between *L. kluyveri* isolates than those in *S. cerevisiae*. This could be due to the absence of a respiro-fermentative lifestyle in *L. kluyveri*, which assigns a more important role to respiration and therefore leads to conservation of expression for this process. The GO categories more conserved at the expression level within these two species are in accordance with those described by Tirosh *et al*. (Translation regulation activity, RNA metabolism,…) (17). This study used a different approach based on expression divergence measured between five *Saccharomyces sensus stricto* species (17) to determine stable and unstable GO categories.

We also observed a higher range of variations among isolates of *L. kluyveri* in comparison with *S. cerevisiae*, which could supposedly be linked to the higher genome sequence diversity found in this species (2.6% compared to 0.6%, respectively). However, sample processing and data treatment were not identical in both studies, which could also explain the differences in range.

**Figure 3.**
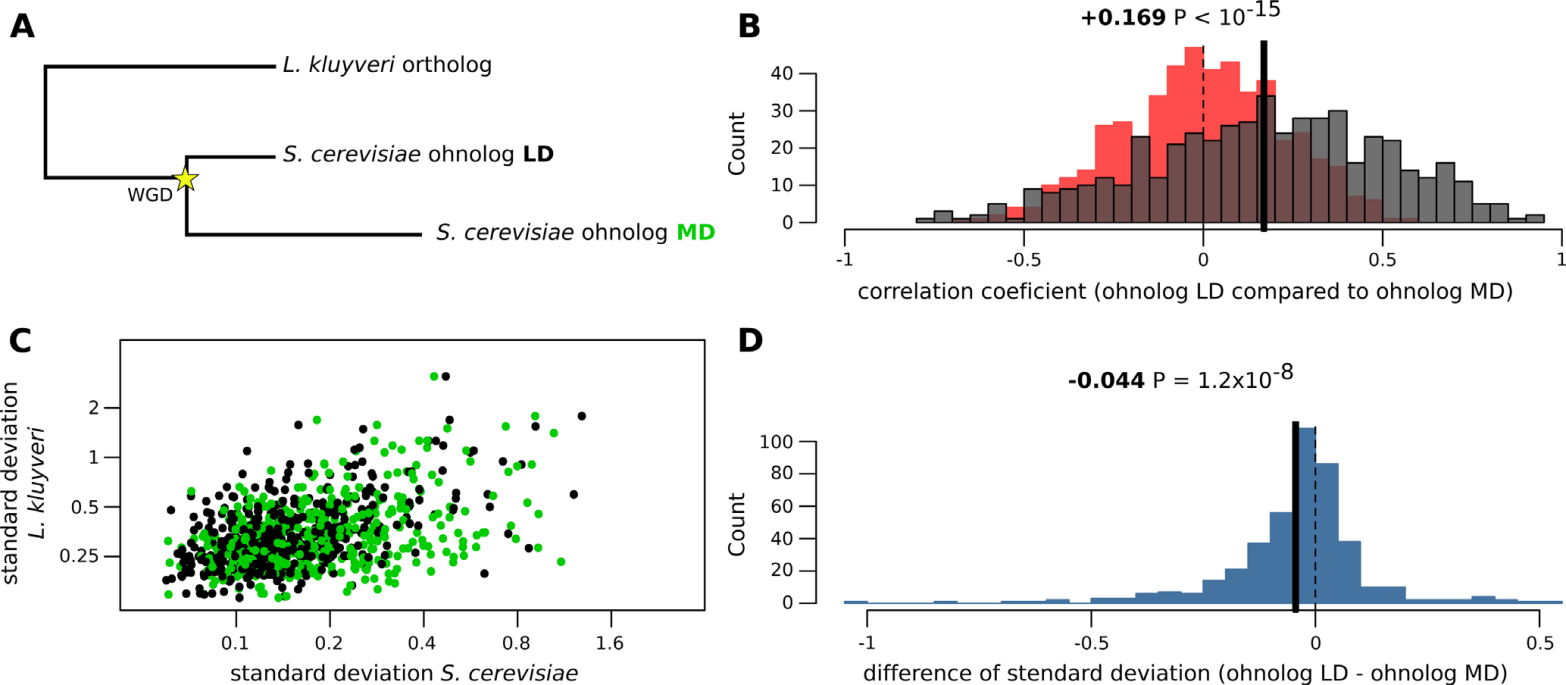
Comparison of expression variation of the 534 ohnolog pairs conserved after the WGD in *S. cerevisiae*, with the corresponding unique ortholog in the protoploid species *L. kluyveri*. (**A**) Scheme depicting the evolution of genes after the WGD, LD: less divergent, MD: more divergent. (**B**) Repartition of the degree of co-expression of the two ohnologs in *S. cerevisiae*. (**C**) Comparison of expression variation of the ohnolog pairs with the *L. kluyveri* orthologs. Black: less divergent ohnologs, green: more divergent ohnologs. (**D**) Repartition of the difference in variability between the two ohnologs.

As *L. kluyveri* is a protoploid species, we also wanted to determine if there was an impact of the WGD on expression variation. Therefore, we compared expression variation of 534 pairs of ohnologs in *S. cerevisiae* that were conserved after WGD, to the expression of their orthologs in *L. kluyveri*. We distinguished the two ohnologs by their similarity levels with the *L. kluyveri* orthologs, in each case one was less divergent (LD) and the other more divergent (MD) (Figure 3A). We first compared expression of both ohnologs across isolates and observed a positive correlation in the majority of case, with 24 highly co-expressed (Spearman correlation > 0.7, no enrichment; Figure 3B). Surprisingly, four of the ohnolog pairs displayed negative correlations (Spearman correlation < −0.7) such as the ADP/ATP translocators *AAC3*/*PET9*, and the phosphoglucomutases *PGM1*/*PGM2* involved in hexose metabolism. In this situation, the variation in expression of one ohnolog compensated for the variation of the other. Therefore, we propose that in these particular ohnologs, the duplication has allowed for correction in expression changes across strains. We then compared the mRNA abundance and standard deviation between the *L. kluyveri* ortholog and the two ohnologs of *S. cerevisiae*, and observed a noisy but positive correlation for both (Figure 3C and Supplementary Figure S1A). However, the noise is lower when these two correlations are made with the LD ohnolog (LD: R^2^ = 0.506; MD: R^2^ = 0.345 for the correlation of expression levels; Supplementary Figure S1A). This indicated that more diverged ohnologs also displayed increased changes in their expression behavior. Therefore, we quantified this difference and observed that, on average, the transcription level for the LD ohnologs is higher than MD ohnologs and, inversely, the standard deviation is lower for the LD ohnologs (Figure 3D and Supplementary Figure S1B). Overall, these observations suggested that, in ohnolog pairs arisen from the WGD, the more conserved gene tends to display the same expression behavior as the ancestor and the gene that is MD displays changes in transcription control. This less conserved gene is likely to demonstrate reduced expression and higher variation among isolates, which is in line with previous observations (32).

### Regulatory networks generate modules of co-expression that change across species

Expression control is organized in modules and regulatory networks resulting in co-expression variation across conditions and genetic backgrounds. We studied expression variation among isolates by a clustering analysis, which consisted of grouping genes that displayed the same variation between isolates. For this analysis, the genes were filtered so that only the relevant clusters were considered (height lower than 3; see the Materials and Methods section). Therefore, 2397 genes out of the 5042 studied were used for clustering (Supplementary Figure S2). We looked for functional enrichment within each cluster. In most instances, we were able to identify enrichment in biological processes, including respiration, lipid and nitrogen metabolism, transcription and ribosome biogenesis (Supplementary Figure S2). We also observed two clusters enriched in genes related to growth rate. Indeed, during RNA sampling, we measured the growth rate of each strain (from 0.52 s^−1^ to 0.23 s^−1^) and determined which genes had expression levels that correlated to this value. We observed 142 positively correlated genes (Spearman coefficient higher than 0.6), which were mainly involved in translation (72 genes, *P* < 10^−14^) and tRNA aminoacylation (nine genes, *P* = 3.3 × 10^−8^). This indicates that cells with high growth rates require increased levels of protein production, which was already observed in *S. cerevisiae* (2,33). Indeed, 27 genes were also identified in the list of 258 genes positively correlated to growth rate proposed by Brauer *et al*. (33), the majority of which (22 genes) correspond to ribosomal proteins. Conversely, there were only 63 genes negatively correlated to the growth rate, 30 of which did not have orthologs in *S. cerevisiae* or were pseudogenes. Therefore, no significant enrichment was found in this group; however, several of the genes are involved in fatty acid metabolism or transmembrane transport and are likely expressed in response to cellular stress associated with membrane renewal. Only seven genes overlap with the 367 genes negatively correlated to growth rate proposed by Brauer *et al*. (33) and were mostly involved in fatty acid metabolism (*FOX2*, *POX1*, *CAT2*). We did not observe enrichment of the genes involved in the oxidation–reduction process or the tricarboxylic acid cycle in the negatively correlated genes in *L. kluyveri*, contrary to what were found in *S. cerevisiae*.

As it was previously shown, the regrouping of genes in functionally enriched, co-expressed modules is common. We performed the same analysis with the *S. cerevisiae* expression data (12) and observed that most clusters were enriched for the same biological processes identified in *L. kluyveri* (Supplementary Figure S3). Across isolates, the co-expression of genes involved in the same pathway indicates that they display strong regulatory links that are not affected by genetic background. However, expression variation in the population is likely to affect whole modules, highlighting complex networks of activation/repression. However, two clusters found in *L. kluyveri*, one involved in protein glycosylation and cell division and another involved in lipid biosynthesis and glycolysis, were not found with the *S. cerevisiae* data set. These differences suggested that, for rare pathways, the strength of conservation of co-expression across isolates changes between species.

The clustering analysis provides a valuable overview of the main modules; however, we wanted to assess the integration of genes in different networks. Therefore, for each gene we counted the instance of positive expression correlations with other gene and used this number as a means to assess the level of expression connectivity. We chose a permissive threshold (Spearman correlation coefficient higher than 0.65) for which no connectivity score higher than 9 could be expected by chance. First, we considered the 782 genes with high connectivity (higher than 150) and observed enrichment for translation (*P* < 10^−14^) and ribosome biogenesis (*P* < 10^−14^), which was expected due to the large number of genes involved in such conserved pathways. The 937 genes that have low/no connectivity (under 10) were mostly genes with no ortholog in *S. cerevisiae* or involved in transport (*P* = 1.9 × 10^−8^). This indicates that the expression of genes involved in the uptake of nutrients and export of molecules is likely to change independent of network, which might prove to be an efficient mechanism for strain adaptation to shifts in ecological niches.

To highlight which regulatory networks are conserved across species, we compared connectivity between *S. cerevisiae* and *L. kluyveri* orthologs (Supplementary Figure S4). As we had already done with the standard deviation, we focused on three types of behavior seen among ortholog pairs. (i) Pairs that are highly connected in both *S. cerevisiae* and *L. kluyveri* (above 100 in both case, 272 genes), which are mainly genes involved in ribosome biogenesis and translation. (ii) Pairs that are highly connected among *L. kluyveri* isolates but not in *S. cerevisiae* (above 100 for *L. kluyveri* and under 15 for *S. cerevisiae*: 295 genes), in which genes are involved in translation (*P* = 2.3 × 10^−5^), protein N-glycosylation (*P* = 7 × 10^−5^) or in pheromone response (*STE5*, *SRM1*, *STE12*, *PRR1*, *STE4*, *STE13*, no enrichment). (iii) Pairs that are highly connected among *S. cerevisiae* isolates but not in *L. kluyveri* (above 100 for *S. cerevisiae* and under 15 for *L. kluyveri*: 155 genes). In this last case, we did not find clear instance of enrichment but observed several genes involved in amino acid biosynthesis (*ADE3*, *ARG2*, *CPA2*, *HIS4*, *LYS14*, *LYS12*, *TRP1*). Interestingly, these results indicated that in *L. kluyveri*, N-glycosylation (i.e. cell wall biosynthesis and cell signaling) is linked to carbohydrate pathways and pheromone response, forming a large module of over 100 genes. On the other hand, between isolates, variation of amino acid biosynthesis is organized in small, less dependent clusters.

### Variation of expression is mainly under complex genetic control

We wanted to assess to which extent the expression of genes is impacted by genetic background. Indeed, as sampling has been completed under the same conditions in all cases, we can assume that the change in transcription was due to the molecular variations. However we also had access to experimental variations by comparing the biological replicates. Therefore, for each gene, we calculated the ratio of the experimental variance over global variance and, from this, obtained the broad sense heritability (H^2^), which represented the portion of expression variation due to genetics.

Most of the transcribed genes (88%) displayed higher heritability than what was expected by chance (H^2^ > 0.7), indicating that the vast majority of gene expression variations are under significant genetic controls. The same observation was previously made for *S. cerevisiae* expression (34). Most of the gene expression that displayed low heritability levels did not vary much among isolates and was involved in mitochondrial translation (*P* = 1.9 × 10^−7^), transcription (*P* = 1.3 × 10^−6^) and glucose transport (*P* = 7 × 10^−5^). Interestingly, these results indicated that the conservation of expression level could be linked to fundamental functions. Therefore, it is possible to seek the genetic marker controlling the majority of gene expression.

To attain an overview of the complexity of genetic control, we determined if the mode of variation of expression followed a bimodal distribution. Indeed, phenotypes following a bimodal distribution between isolates are likely under a major allele control, where only one gene is affected by a mutation in the collection, with a balanced repartition. Conversely, variation controlled by mutations distributed among different genes will display a more unimodal variation. We ran a *k*-means model (*k* = 2) on the mRNA levels for each gene. Only 30 genes displayed a variation of expression in a bimodal shape in the population, among which were found those involved in mating (*STE2*, *STE3*, *STE14*, *SAG1* orthologs) and galactose metabolism (*GAL7* and *GAL10* orthologs). Six of these genes do not have orthologs in *S. cerevisiae*. This low number of genes displaying a bimodal repartition indicated that the expression variation for most of the genes followed unimodal or multimodal patterns. This demonstrated that genetic controls on expression are, in the majority of cases, complex with multiple markers involved and almost no genes are under clear major allele controls, apart of the usual *MAT* or *GAL* loci.

### Protein conservation and expression behavior are linked to gene function

We then wanted to determine if there was a link between expression level or variation and gene conservation, i.e. the apparition of consistent mutations. We calculated the ratio of non-synonymous (dN) to synonymous (dS) substitution rates (dN/dS) for each gene. This ratio indicates if the gene is under purifying (dN/dS < 1) or positive selection (dN/dS > 1). Across all strains, we observed only 23 genes with an average dN/dS greater than 1, with only one that has an ortholog in *S. cerevisiae*, *CYC2*, a mitochondrial membrane protein involved in cytochrome C maturation (35). While most genes were found to be under purifying selection, we observed variation in the strength of this selection. About 120 genes display an average dN/dS equal to zero, indicating that no non-synonymous mutations were found within the population. These genes are mainly involved in translation and ribosome biogenesis (*P* < 10^−14^), or are a constituent of mitochondrial cytochrome C (*P* = 4 × 10^−5^). The analysis also revealed that 167 genes tend to have less pressure for conservation of the protein sequence (dN/dS > 0.4); however, no functional enrichments were found as most of the corresponding orthologs have an unknown function (*COS3–8*).

**Figure 4.**
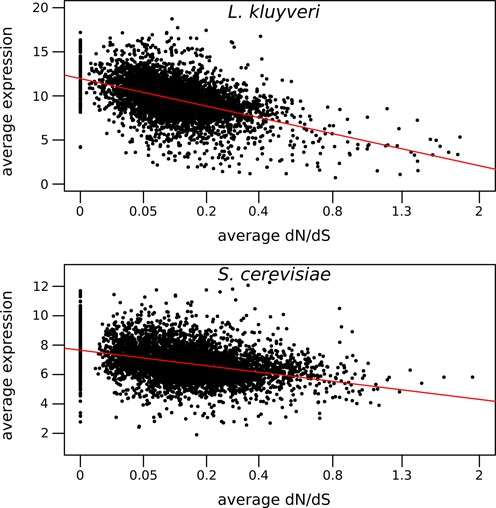
Relationship between expression level and dN/dS in *L. kluyveri* (top) and *S. cerevisiae* (bottom). The dN/dS axes are in a square root scale. The lines correspond to linear regression (*L. kluyveri*: R^2^ = 0.234 *P* < 10^−16^; *S. cerevisiae*: R^2^ = 0.146 *P* < 10^−16^).

We compared expression proprieties to the dN/dS and observed a negative correlation between transcription levels and dN/dS (Figure 4). The same observation was made for *S. cerevisiae* by using the publicly available sequences and expression data (12). Genes that are highly expressed, such as the *TDH2* (glycolysis) ortholog or the *HTB1* (core histone protein) ortholog, tend to display low dN/dS values while genes with weak expression, such as the *TEN1* (regulation telomeric length) ortholog or the *ATR1* (detoxification) ortholog, have a higher dN/dS. This relationship is likely to be due to gene function. Genes that are part of the core genome or with an essential role in fundamental pathway display generally high levels of transcription. Mutations in such proteins will have fitness drawbacks and therefore disappear rapidly. On the other hand, low or conditionally expressed proteins can accumulate consistent mutations, to a certain extent, as the dN/dS values are still below 1.

### Evolution of the gene expression pattern of in large (1 Mb) introgressed region

**Figure 5.**
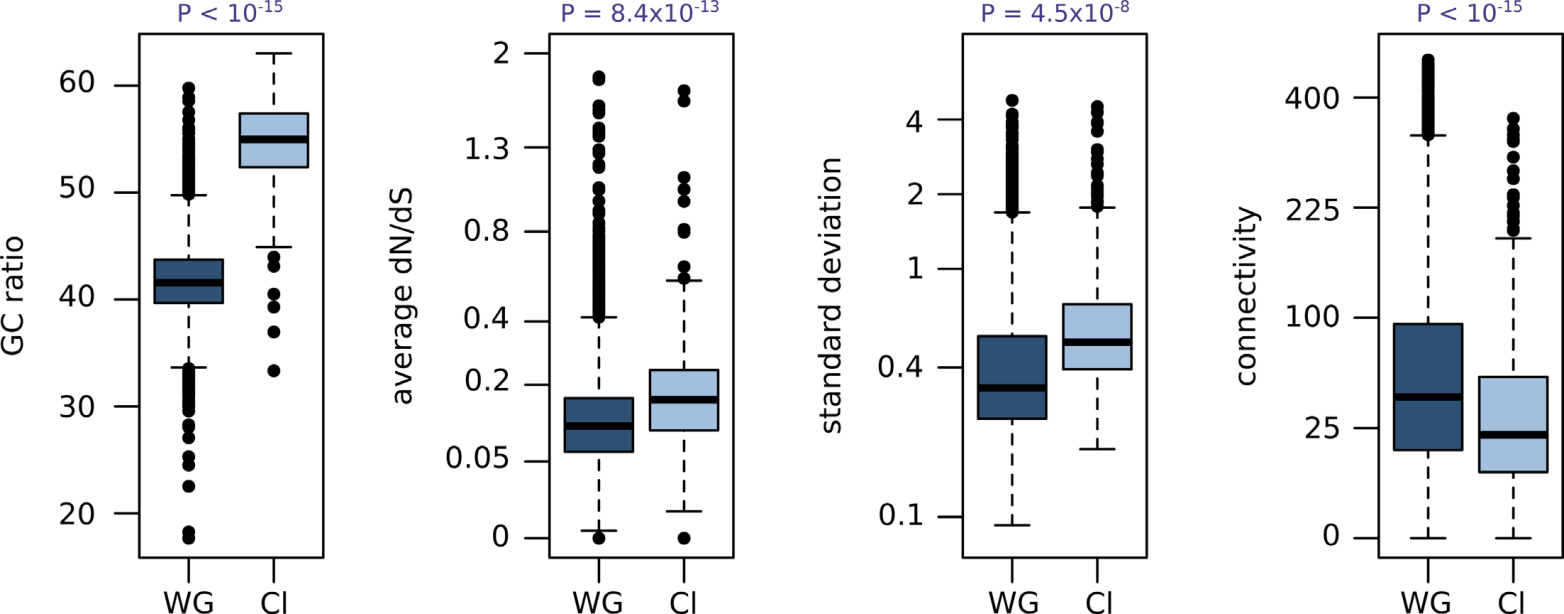
Comparison of gene characteristic and expression behavior between the Sakl0C-left and the rest of the genomes, WG: whole genome, Cl: Sakl0C-left. Standard deviation axis is in a log scale, connectivity and dN/dS axes are in a square root scale.

An additional unique characteristic of *L. kluyveri* is the large introgression found on the left arm of chromosome C (1 Mb in length hereafter called Sakl0C-left). This region has a surprisingly high GC content and our previous works showed it also harbors a higher recombination rate and a substitution rate biased toward G/C bases, leading to the conclusion that this left arm has a distinct evolutionary history in comparison with the rest of the genome (24). The higher GC ratio includes the intergenic and coding regions (Figure 5). We compared the dN/dS of the 468 genes in this specific region (for which we have expression measurement) to the rest of the genome. Even if almost all the genes were under purifying selection (dN/dS < 1), they tended to display higher dN/dS values, with an average increase of 0.06 (*t-*test *P* = 8.4 × 10^−13^; Figure 5).

We then compared the stability of the transcription level of the Sakl0C-left genes to the whole genome and observed a general increase in the standard deviation (*t-*test *P* = 4.5 × 10^−8^). This indicated that gene expression, in this particular region, is more sensitive to genetic variation. We compare the connectivity of the genes on the Sakl0C-left to the whole genome to observe if they are involved in large regulatory networks. We found a significant reduction in the connectivity for these genes, with an average of 31.6 compared to 53.6 for the whole genome (*t-*test *P* < 10^−15^; Figure 5).

These different results indicated that genes of the Sakl0C-left are under lower purifying selection pressure and their transcription dramatically varies among isolates. These genes are less integrated in modules of co-expression with fewer links to the regulation of other genes. This observation confirmed the difference in evolutionary history of this large chromosomal region, suggesting that these genes are not yet completely integrated into the *L. kluyveri* networks and display more plasticity in their expression and protein sequence than the rest of the genome.

### Gene annotations based on conservation can be expanded through expression pattern

**Figure 6.**
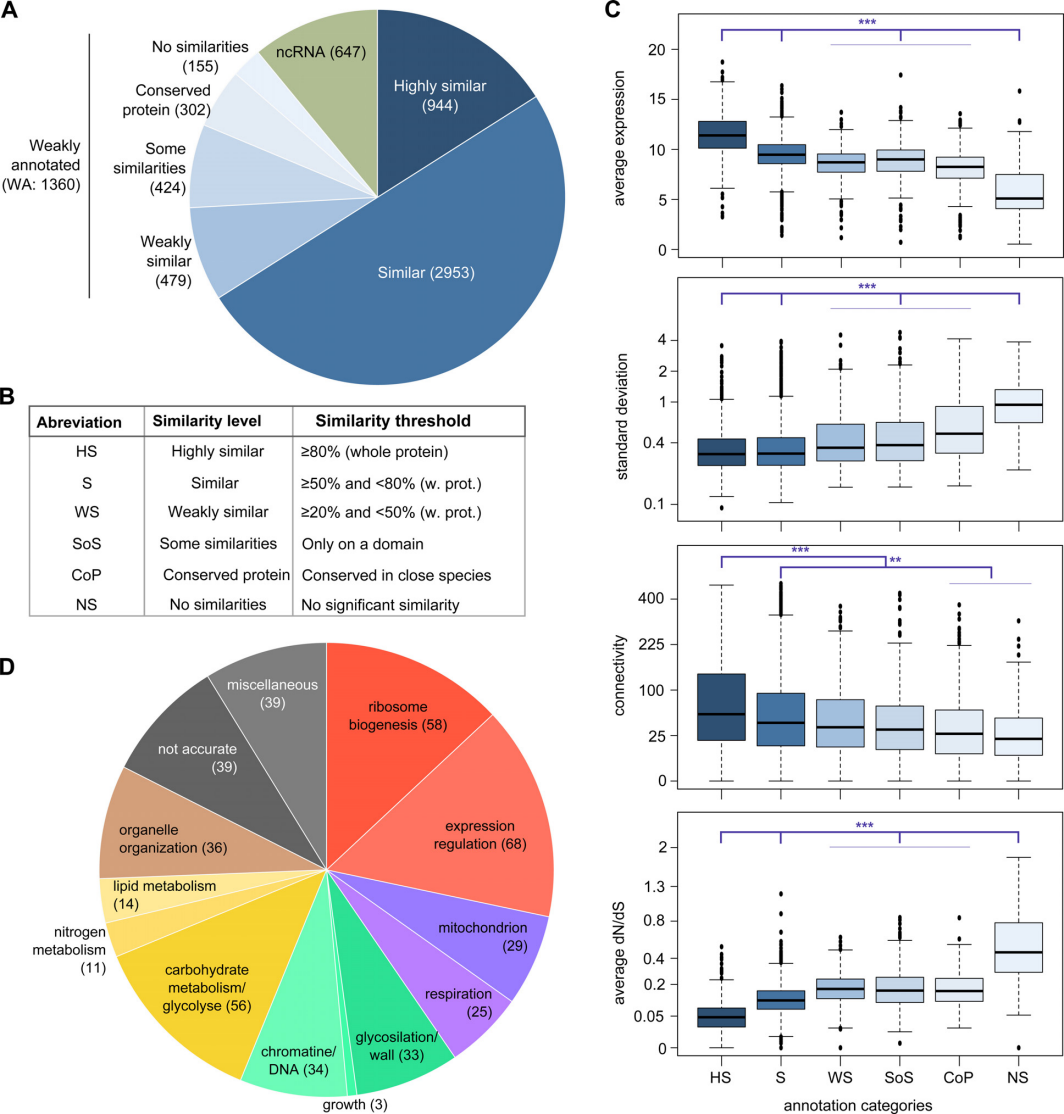
Functional annotation of the *L. kluyveri* genome. (**A**) Functional annotation of the *L. kluyveri* genome based on homology displayed as a pie chart of categories of similarity levels with the ortholog. (**B**) Correspondence table of categories with similarity thresholds. (**C**) Gene expression characteristics as a function of the similarity level with known homologs, displayed using box plots. Expression levels are given in log2 (normalized read number), standard deviation axis is in a log scale, connectivity and dN/dS axes are in a square root scale. Statistical comparison was performed by a Tukey test, which allowed multiple comparisons (***P < 10^−6^, ** *P* < 10^−3^). (**D**) Pie chart of the biological processes superfamily corresponding to the assigned GO term found for the 445 weakly annotated genes (WS, SoS, CoP and NS).

As gene annotation in *L. kluyveri* is based on orthologs in *S. cerevisiae*, the reliability of this annotation depends on how well the genes are conserved between these two species. We distinguished six categories of annotation. For four classes, orthologs were found in *S. cerevisiae*: highly similar (HS), similar (S), weakly similar (WS) and some similarity in domains (SoS). Additionally, two classes were used to describe instances when no *S. cerevisiae* orthologs were observed: conserved proteins (CoP) found in related species and proteins with absolutely no similarity (NS) in other species (Figure 6A and B).

We wanted first to determine if gene conservation is linked to expression behavior. By studying the average expression level among genes, we observed that highly conserved genes (i.e. with a similarity level to the *S. cerevisiae* ortholog higher than 80%) are also well expressed (Figure 6C) and correspond mostly to the core genome (genes conserved across species and involved in fundamental biological process, i.e. mitochondrial translation, ribosome biogenesis). On the contrary, the genes unique to this species display low levels of expression. A number of these genes could be dubious and not actually transcribed, however, as we performed this analysis in only one growth condition, we must acknowledge that these genes might be under conditional activation. Across isolates, transcription variation was lower for well-conserved genes while those specific to *L. kluyveri* displayed very high levels of variation (Figure 6C). Genes conserved between species also demonstrate more conservation in protein sequence at the intraspecies level, with very low dN/dS values. Strikingly, genes only found in *L. kluyveri* displayed very high dN/dS values, with 22 of them greater than 1. We also discovered a positive link between similarity level and connectivity, indicating that genes conserved across species are more likely integrated in co-expression modules (Figure 6C).

Consequently, we detected a strong link between gene conservation and expression behavior. Well-conserved genes display higher expression levels, less variability in transcription and protein sequence and are more likely involved in networks. On the other hand, genes specific to *L. kluyveri*, of which 25% are located in the Sakl0C-left region, tend to display more transcriptional variation.

When inspecting the annotation, we found that 23% of the genome presented few or no similarities with *S. cerevisiae* genes and therefore are weakly annotated (WA). Furthermore, there are 155 genes unique to this species (NS category). Therefore, enhancing the genome annotation using a high throughput analysis for *L. kluyveri* is crucial. We sought to use the connectivity data to propose, for each gene, a biological process in which it might be involved. Indeed, the clustering analysis demonstrates co-expression of genes that are involved in the same biological processes. From the connectivity analysis, we obtained, for every genes, the list of positively correlated genes that also have reliable annotations and then used *S. cerevisiae* orthologs to look for biological process GO term enrichment.

Among the 4467 genes that had enough positively correlated genes, 2265 revealed significant enrichment (50%, *P* < 10^−4^). In agreement with the higher connectivity, our approach provided a high rate of putative process for genes that are highly similar to their *S. cerevisiae* orthologs (66%) while the genes specific to *L. kluyveri* had lower rates of successful output (19%).

Considering all our results, we proposed putative GO terms for 36% of the genes that have no reliable annotation (WA) (Figure 6D). The processes identified are biased by the over-representation of genes involved in large modules, such as ribosome biogenesis. However, we were able to provide putative processes in various domains such as expression, cell growth and metabolism. Concerning the genes specific in *L. kluyveri*, 24 demonstrated significant enrichment (Supplementary Table S1), while the remaining 126 were not associated with any GO term. However, since they are not conserved across species, they could be non-functional proteins. The reliability of this approach is difficult to assess, nevertheless these data could provide information for future functional analysis in *L. kluyveri*.

## DISCUSSION

In budding yeast, it has been shown that *S. cerevisiae* is not always an ideal representative of other species, partly due to the significant impact of human domestication on the population structure. Here, we provide a thorough description of transcription variations in a population of *L. kluyveri* natural isolates. Applying this type of approach to a protoploid species allowed us to compare the evolution dynamics of gene expression between distantly related species. Our data helped identify genes that displayed coordinate variation (4250 genes with connectivity higher that 15) as well as those with independent regulation (937 genes with connectivity lower than 10). The clusters, or modules of co-expressed genes, can be linked to specific biological processes. This global organization of regulatory networks is highly conserved across species, with intrinsic changes that can be followed using interspecies studies and also linked to metabolic evolution (21). Here, we have described specific *L. kluyveri* regulatory networks with a link found to the glycosylation and pheromone responses, and with dissociation from the amino acid biosynthetic pathways. Using this clustered structure we proposed putative biological process for uncharacterized genes. However, this approach should be complemented by an analysis of transcriptomic response to environmental change.

Using *L. kluyveri*, we had the opportunity to observe the evolution of transcriptomic control in three different contexts of genome evolutionary history. First, we analyzed a species that has not undergone the WGD, allowing the description of differential intraspecific expression variations between ohnologs through comparison to the original ortholog. Second, we revealed that the acquisition of a large introgression in the ancestor of this species resulted in specific expression behavior. Third, *L. kluyveri* encodes many unique genes that display transcriptomic signs of their recent acquisition. In each case, we linked the evolutionary dynamics of gene expression, to the ultimate goal of increasing gene fitness in the species networks. Therefore, expression levels appear to be lower, with a higher variability across isolates and less coordination in modules. Moreover, mutations that could affect proteins function appear to be more frequent in such genes. These observations reveal dynamics in gene evolution, which are in agreement with previous studies, in yeast (32,36,37), as well as other organisms (38,39). However, the particular characteristics of *L. kluyveri* allowed us to clearly highlight the transcriptomic signature of genes not yet well integrated in the genome and regulatory networks.

## SUPPLEMENTARY DATA

Supplementary Data are available at NAR Online.

SUPPLEMENTARY DATA
