# Supplementary material for: Evolution of intraspecific transcriptomic landscapes in yeasts
Source: Nucleic Acids Res. 2015 Apr 20;43(9):4558–68. doi: 10.1093/nar/gkv363 (PMC4482089; doi:10.1093/nar/gkv363)
Supplement: SUPPLEMENTARY DATA [file supp_43_9_4558__index.html]

Evolution of intraspecific transcriptomic landscapes in yeasts — Evolution of intraspecific transcriptomic landscapes in yeasts — SUPPLEMENTARY DATA 

# Evolution of intraspecific transcriptomic landscapes in yeasts

## SUPPLEMENTARY DATA

**Files in this Data Supplement:**

- SUPPLEMENTARY DATA
