# Supplementary material for: Evolution of intraspecific transcriptomic landscapes in yeasts
Source: Nucleic Acids Res. 2015 Apr 20;43(9):4558–68. doi: 10.1093/nar/gkv363 (PMC4482089; doi:10.1093/nar/gkv363)
Supplement: SUPPLEMENTARY DATA [file supp_gkv363_nar-00513-z-2015-File008.pdf]

Supplementary Information

Evolution of intraspecific transcriptomic landscapes in yeasts

Christian Brion, David Pflieger, Anne Friedrich and Joseph Schacherer

Supplementary figure S1

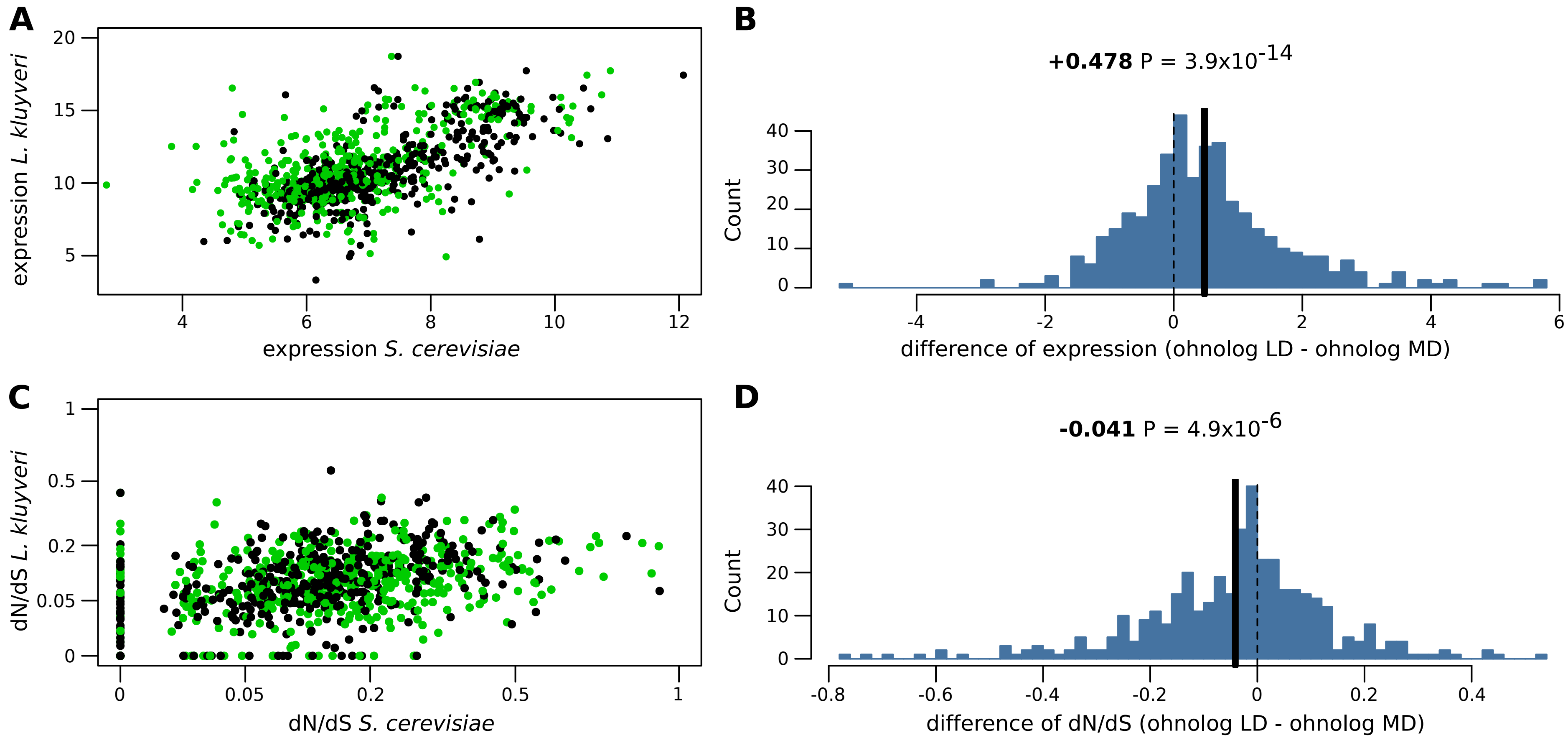

Comparison of expression variation of the 534 paralogs pairs conserved since the WGD in *S. cerevisiae*, with their unique otholog in the protoploid species *L. kluyveri*.

A Comparison of expression levels of the paralog pairs with the *L. kluyveri* ortholog. black: less divergent paralogs, green: more divergent paralogs. **B** Repartition of the difference of expression between the two paralogs. **C** Comparison of the dN/dS of the paralog pairs with the *L. kluyveri* orthologs. black: less divergent paralogs, green: more divergent paralogs. **D** Repartition of the difference of the dN/dS between the two paralogs.

Supplementary figure S2

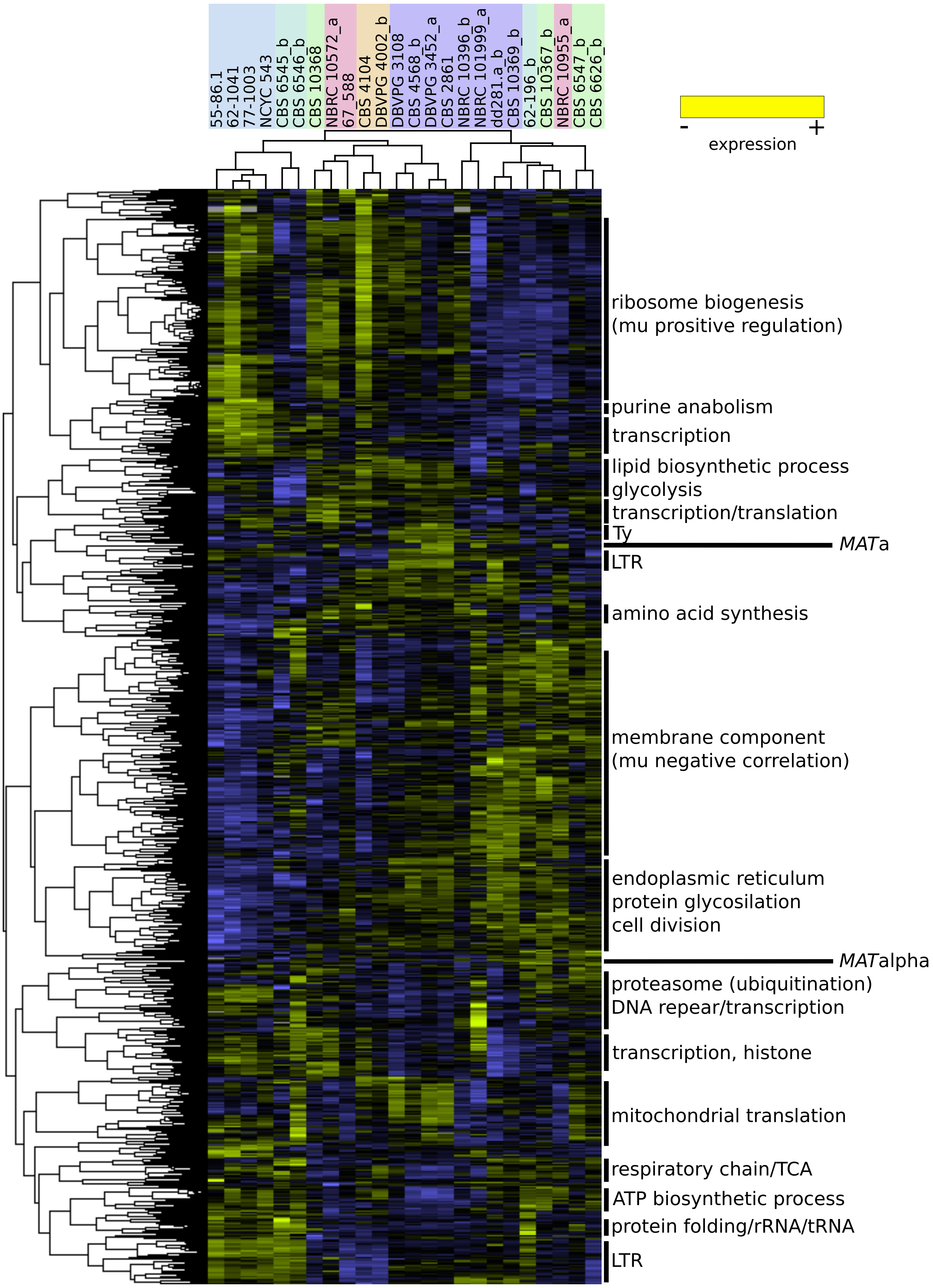

Clustering analysis of intraspecific expression variation

Clustering analysis of expression variation across the 24 strains of *L. kluyveri* and the 2675 genes displaying significant first level clusters (height lower than 3, hclust R function). GO term enrichment analyses were performed using S. cerevisiae orthologs with FunSpec (Robinson et al. 2002).

Supplementary figure S3

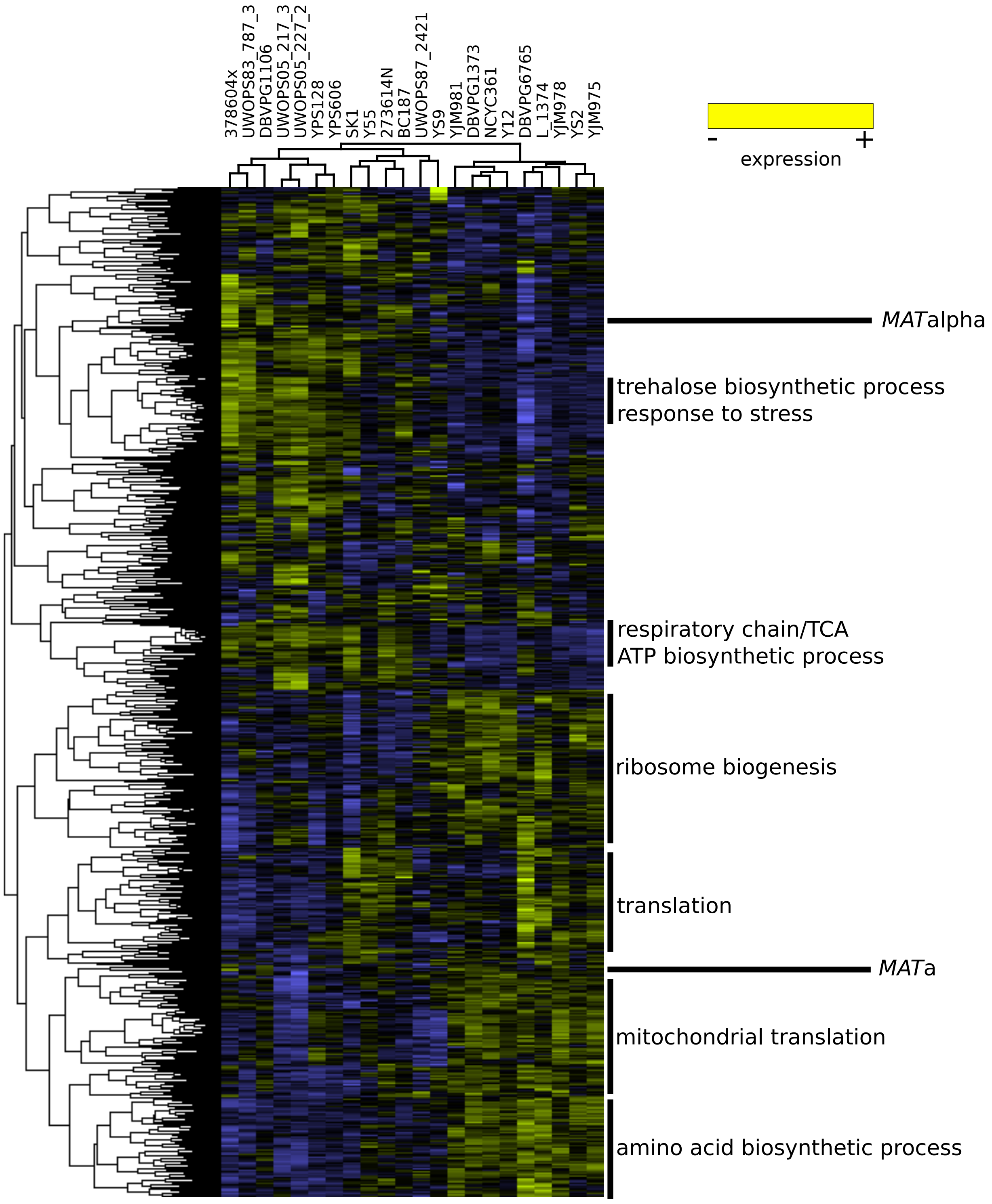

Clustering analysis of expression variation in *S. cerevisiae*

Clustering analysis of expression variation across the 22 strains of *S. cerevisiae* and the 2681 genes displaying significant first level cluster (hight lower than 3, hclust R function). GO term enrichment analyses were performed using FunSpec (Robinson et al. 2002).

Supplementary figure S4

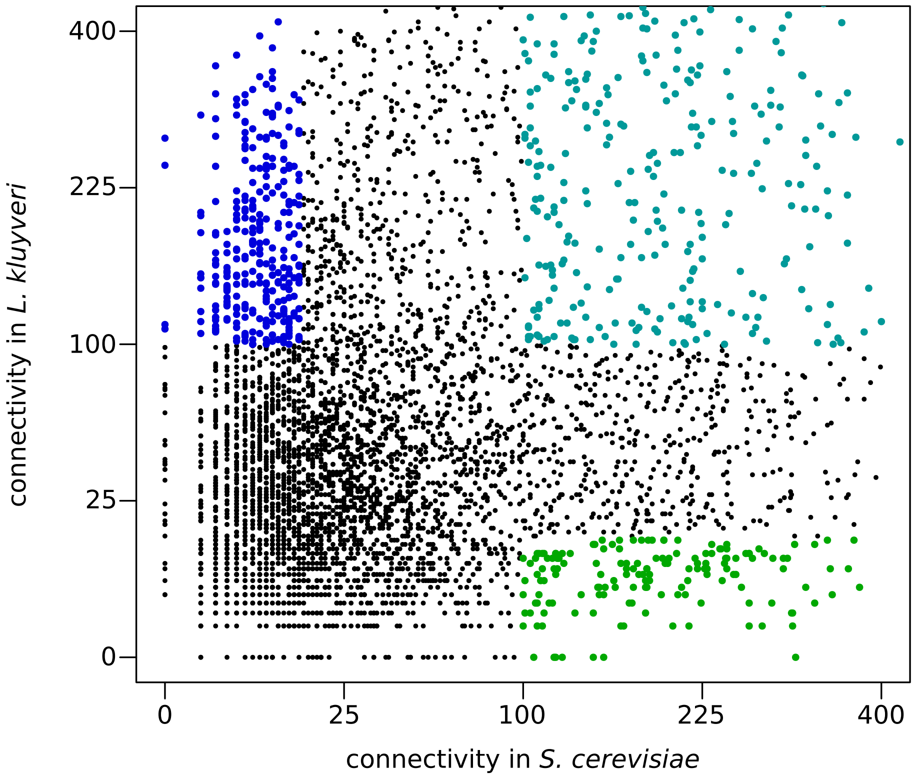

Comparison of connectivity between *L. kluyveri* and *S. cerevisiae* orthologs.

Colors correspond to the sets of genes we used for enrichment to highlight transcriptomic difference between *L. kluyveri* and *S. cerevisiae*. The connectivity axes are in a square root scale.

Supplementary table S1: List of *L. kluyveri* specific genes for which a biological process has been proposed

Genes	Average expression level	Standard deviation	dN/dS	Number of genes used for the enrichment	P-value	Associated biological process GO term
SAKL0A02706g	5.86	2.00	0.28	20	1.81E-06	lipid catabolic process
SAKL0A08646g	7.24	0.22	1.43	155	7.48E-05	oligosaccharide biosynthetic process
SAKL0B03190g	9.55	0.60	0.52	122	3.45E-09	protein glycosylation
SAKL0B04048g	5.87	0.36	0.44	40	8.19E-05	polysaccharide catabolic process
SAKL0C01298g	15.83	0.32	0.05	27	2.32E-08	tricarboxylic acid cycle
SAKL0C02530g	4.84	0.35	0.40	163	1.36E-05	carbohydrate metabolic process
SAKL0C05852g	7.58	0.23	0.57	72	1.25E-06	protein glycosylation
SAKL0C11638g	6.10	0.47	0.57	96	1.95E-18	ribosome biogenesis
SAKL0D03080g	7.91	0.44	0.33	92	1.56E-47	ribosome biogenesis
SAKL0D09790g	6.31	0.34	0.63	36	1.70E-05	polyol catabolic process
SAKL0E04807g	8.38	1.05	0.25	39	2.15E-05	protein retention in ER lumen
SAKL0E11726g	6.62	0.30	0.80	36	4.22E-06	regulation of DNA replication initiation
SAKL0E12276g	5.27	0.27	0.11	7	2.79E-05	tetrahydrofolate biosynthetic process
SAKL0F00176g	2.17	0.20	0.64	57	4.05E-05	protein glycosylation
SAKL0F01232g	3.47	1.63	0.72	37	5.65E-05	nucleobase transport
SAKL0F01936g	9.87	0.42	0.12	32	4.12E-07	DNA-templated transcription
SAKL0F06116g	4.86	0.46	0.71	40	3.98E-07	ribosome biogenesis
SAKL0F11264g	10.72	0.51	0.20	39	4.10E-18	mitochondrial translation
SAKL0F14256g	3.43	0.20	0.42	133	6.58E-07	single-organism catabolic process
SAKL0F14300g	6.63	0.48	0.78	215	1.04E-06	carbohydrate metabolic process
SAKL0H09702g	8.26	1.06	0.09	89	1.44E-06	protein glycosylation
SAKL0H13178g	9.56	0.28	0.56	5	1.33E-05	tryptophan biosynthetic process
SAKL0H14366g	7.97	0.52	0.28	99	3.35E-08	carbohydrate metabolic process
SAKL0H21340g	9.41	0.52	0.45	9	1.07E-09	glycolysis
